# High-intensity focused ultrasound plus concomitant radiotherapy: a new weapon in oncology?

**DOI:** 10.1186/2050-5736-1-6

**Published:** 2013-05-01

**Authors:** Giovanni Borasi, Giorgio Russo, Filippo Alongi, Alan Nahum, Giuliana Carmela Candiano, Alessandro Stefano, Maria Carla Gilardi, Cristina Messa

**Affiliations:** 1 University of Milano-Bicocca, Milan 20126, Italy; 2 IBFM CNR-LATO, Cefalù, Palermo, 90015, Italy; 3 Istituto Clinico Humanitas, Milan, 20089, Italy; 4 Clatterbridge Cancer Centre, Bebington, CH63 4JY, UK; 5 IBFM-CNR, Segrate, Milan, 20090, Italy

**Keywords:** HIFU, EBRT, HT, Integration of HIFU and EBRT, A new weapon against cancer

## Abstract

The potential impact of high-intensity focused ultrasound (HIFU) to general medicine and oncology seems very high. However, while in the research area, the development of this technique is very rapid and unchallenged. The direct application of HIFU to human tumour therapy is hampered by various technical difficulties, which may confine its role to a marginal device in the surgery armamentarium. To deploy the full potential of focused ultrasound in oncology, it seems necessary to review the basic relationship between HIFU and external beam radiotherapy. This is the aim of the present work.

## Introduction

Let us introduce a future scenario, involving the integration of external beam radiotherapy (EBRT) and high-intensity focused ultrasound (HIFU), with a case history taken from our collection of bone metastasis treatments using magnetic resonance-guided high-intensity focused ultrasound (MRgFUS) [1].

The patient (female, 55 years old, with pelvic metastases from rectal adenocarcinoma) spent more than 3 h in the MRI machine undergoing MRgFUS treatment by the medical team with an Exablate 2100 (InSightec, Tirat Carmel, Israel [2]) for the pain palliation of a fairly large pelvic bone metastasis. At that point, there was no reason to continue: the periosteal innervation, the probable cause of the pain [3,4], had been ablated as well as a large part of the cancerous metastatic tissue. However, the whole metastatic tissue was not destroyed for several reasons: the patient could not endure an even longer stay in the magnet, a part of the potentially cancerous tissue was ‘shadowed’ by a compact bone structure, another (smaller) part was very near to the rectum, and there was no other viable beam direction which would have allowed the ablation to continue. Sometime after the treatment, the patient experienced an extraordinary improvement in his quality of life: the pain almost completely disappeared. The pain interference scale score [5] decreased from 6 to 0.6 and the VAS score [6] from 9 to 3. The patient was no longer bed-bound, and the amount of opiates prescribed was significantly reduced. Three months later, by comparing the new fluorodeoxyglucose (FDG)-PET/CT examination with that done before the MRgFUS treatment, the images left no doubts. The ablated region did not show any FDG uptake, but where we were not able to ablate, a considerable, non-painful, tumour proliferation was evident. At that point, it was clear to anyone that the best treatment would have been HIFU plus concurrent radiotherapy.

It can be added that according to current protocols, HIFU is only employed for palliative treatment of bone metastases (pain reduction), when the disease has become systemic. In several cases, complete success (pain palliation and total metastasis ablation) has been obtained [7]. However, in the above case history, would there have been a realistic chance for EBRT to have been effective? Even if the management of bone metastases requires a multidisciplinary approach [8], the answer is that local EBRT is probably the treatment of choice for localised bone pain [9-11]. There is a large body of published reports that confirms the efficacy of this treatment, in particular using the new highly conformal treatments. Overall, 70–80% of patients will respond, and up to one third will achieve a complete response [12]. The HIFU challenge is to do even better, and there is increasing evidence in this direction [13-16]. This particular patient was first proposed for EBRT, but he preferred HIFU. Therefore, in locations where HIFU was impossible to administer, EBRT would have been the best choice. Another way could have been to employ one of the minimally invasive techniques (like alcohol injection or LASER, or radio-frequency/microwave ablation or cryotherapy). These techniques are a very important resource in the management of bone metastases [9]. In this particular case, due to the lesion dimensions and the presence of bone and sensitive structures (rectum, nerves), the above techniques would encounter severe difficulties in the ablation of the whole of the cancerous tissue. EBRT, which can easily penetrate the thick bone structure, can reach and, hopefully, sterilize each part of the lesion and even guarantee a safety margin around the tumour.

### HIFU, hyperthermia and radiotherapy

In our opinion, there is no scientific rationale for trials comparing the palliative effect of MRgFUS versus EBRT in bone metastasis treatment. In fact, the practicalities of implementation and the physical and biological mechanisms are totally different. Provided that HIFU can be administered (given constraints such as nerves, bowel and non-targeted bone in the beam path [17]), it directly destroys the periosteal innervation, the probable cause of the pain [3,4], thereby resulting in a remarkable and enduring pain reduction [13-16]. On the other hand, in some cases it cannot eradicate the whole cancerous tissue. Radiation has fewer constraints and acts by reducing (or eliminating) the proliferative effect of the tumour. Pain reduction may result from lowering the pressure of the tumour on the nerves of endosteum or on nerve roots inside the lesion [3]. In addition, EBRT can treat larger targets though it may be less effective in pain palliation. In fact, less than 30% of the patients experience complete pain relief [12], and also this is frequently temporary with recurrence of pain in 57% of patients at 15 weeks (median value) after completion of the treatment [18]. Being highly complementary, the combination of EBRT and HIFU could, in many cases, provide the patient with the best possible treatment. The same may be true for tumours other than bone metastases.

### A key question arises: what is the optimal way to use HIFU plus EBRT and what is the best timing for combining them?

Photon EBRT has more than a hundred years of history, and consequently, a great deal is known about it. One point seems to be clear: when the tumour has become quite large, the central part is somewhat compressed by angiogenesis, has a poorer blood supply and tends to become hypoxic. In these conditions, ionizing radiation loses much of its destructive capability, which relies on a good oxygen supply [19]. That seems exactly the perfect target for HIFU, whose effect is not significantly dependent on the oxygen supply. On the other hand, the peripheral regions of the tumour, where the oxygen supply is good but also cell proliferation is high, are certainly a better target for radiation, and a good sterilization of quite large volumes may be possible in a reasonable time. The precise localization of the hypoxic region inside the tumour, recently obtained with contrast MR, opens the door to personalized treatments [20]. Also, the optimal timing seems to have a quite simple rationale: when we treat with HIFU, a lot of heat is produced and diffuses from the focus toward external tissue regions, while the temperature progressively decreases. It is well known that mild hyperthermia (HT) increases the blood supply which can make ionizing radiation more effective. A nice example comes from the treatment of primary, locally advanced, cervix cancer, where the beneficial effect of adding HT to EBRT (thermoradiotherapy) has been confirmed in a large population (378 cases) [21]. Of course, the positive effect of HT does not stop immediately when the heated tissues return to the normal body temperature. The effect may last sometime, depending on several conditions, and this could explain the good results obtained from combining HIFU with EBRT, even at intervals of several hours [22]. However, if we could choose the optimal timing, we would say: ‘EBRT immediately after HIFU’ [23,24]. In this way, the heat produced by HIFU, in a time of just a few minutes, would become a powerful enhancer for the concomitant EBRT. The same heat could also be of fundamental importance if we consider the possibility of heat (mild HT)-mediated drug delivery [25]. A more complex recent experiment [26] involved (1) pulsed ultrasound (pFUS), (2) EBRT, (3) an antitumor drug (Docetaxel) and combinations (1) + (3), (2) + (3) and (1) + (2) + (3) on mice bearing prostate tumours. Quite reasonably, the combination of all the three weapons gave the best tumour control. Quite inexplicably, (1) + (2) was not tested. However, considering here mainly HIFU and EBRT, this strategy would allow also a great sparing of time and of radiation dose. A lower radiation level means, in turn, a potentially significant reduction of sequelae, as reported in a recent paper [27]. Of course the scenario described above would require a great deal of fundamental and experimental research. To find, for each tumour type, the best combination of HIFU and EBRT will be as fascinating as that of drug delivery (and it may be possible to do it in the same environment). However, by working with cells or small animals, a sufficient degree of understanding of the HIFU + EBRT phenomenon could be eventually gained without new devices and great financial investment. For larger animals and for humans, however, the problem will be substantially different. The current designs of ‘total body’ HIFU systems (both with US and MR guidance), with an unsealed transducer positioned under the patient couch, requiring a considerable adaptation of the patient to the machine, seem more ‘proof of concept’ than mature systems, specifically oriented to intensive patient cure.

### A new weapon

A new generation of total body HIFU systems may rely, in our opinion, on a sealed phased array transducer, robotically moved in different (anterior and posterior) regions of the patient (or, eventually, on multiple transducers). A first example of this kind is the flat, 1024 piezoelectric element, phased array transducer named ‘Conformal Bone System’ developed by Insightec [2] for treating bone metastasis. This ‘Work in Progress’ probe includes a built-in skin cooling system, based on a water bag and a water-permeable membrane, providing the acoustic coupling. The probe is held manually by the clinician and positioned in different body regions without moving the patient on the couch. Chinese scientists are also producing sealed multi-element transducers that can be coupled to the patient with a water-filled bladder [22]. In the last years, MR-compatible robots have been developed for MR-guided surgery [28] and have been modified to move an HIFU transducer within the MR bore [29,30]. Following this line of thought, we could have not just better clinical HIFU systems, but also systems whose integration in an EBRT environment would be less problematic. A giant step in the integration of systems that appeared to be incompatible was taken by Elekta (Stockholm, Sweden) [31] with the project of a short 6-MV linear accelerator (linac), rotating around a modified MR magnet. This environment would be ideal for integrating also a robotically controlled HIFU transducer. However, also conventional linacs could be provided with robotically managed HIFU systems, using for imaging and temperature control an appropriate echographic system (eventually integrated in the same probe). The great progress in this latter field may make MR guidance non-essential [32-35]. Up to this point, we have focused on body applications, but we cannot forget the problem of brain pathology. To the best of the authors' knowledge, the only HIFU equipment currently available is a dedicated helmet in an MR-guided environment [36,37]. These kinds of systems are opening up new avenues in direct tumour ablation [38] or in localized small-area brain ablation for debilitating pathologies [39]. They are also giving rise to great hopes for localized drug delivery via brain–blood barrier reversible openings [40]. This kind of environment could make direct integration with EBRT difficult, even if, in principle, the patient could be moved on the same table between the HIFU helmet and the EBRT area within the modified MR system we mentioned above [31]. However, robotically guided short linacs are currently used for radiosurgical therapy in the brain [41]. However, even with these highly sophisticated approaches, treatment sequelae are non-negligible [42]. To enhance the treatment efficacy and lower the risks, this robotic environment could be improved by including additional arms, carrying the HIFU transducer and, eventually, an echographic probe. Summarizing, hyperthermia, eventually generated by ablation, is a potent enhancer of EBRT. What is lacking is a single integrated system for concomitant EBRT and HIFU for both the human brain and body.

## Conclusions

The technical and economic difficulties of developing such a device should not be underestimated. On the other hand, we could have a potent new weapon against cancer (and, thanks to drug delivery, also against many degenerative diseases) integrating, in real time, target and temperature imaging, radiation sterilization, hyperthermia, ablation and drug delivery. In all probability, this device would be a lot less expensive and, for many cancer localization, a great deal more effective than expensive and, therefore, practically inaccessible proton or heavy ion radiotherapy.

## Abbreviations

EBRT: External beam radiotherapy; FDG: Fluorodeoxyglucose; HIFU: High-Intensity Focused Ultrasound; HT: Hyperthermia; MRgFUS: Magnetic resonance-guided focused ultrasound.

## Competing interests

The authors declare that they have no competing interests.

## Authors’ contributions

GB studied the concept and design. GR, GC and AS gave basic contributions to the HIFU physics. FA is clinically responsible for the radiotherapy treatments. AN gave fundamental contributions to the radiotherapy and radiobiology theoretical foundations. CM performed the clinical interpretation of the nuclear medicine images. MCG supervised the study. All authors read and approved the final manuscript.
